# High Doses of *N*‐Acetyl‐*P*‐Aminophenol Cause Acute Changes in Electroencephalographic and Electrocardiographic Activities in Wistar Rats

**DOI:** 10.1111/bcpt.70321

**Published:** 2026-10-08

**Authors:** Alex Luiz Menezes da Silva, Luiz Gustavo dos Reis de Freitas, Akira Otake Hamoy, Clarissa Araujo da Paz, Luan Oliveira Ferreira, Esther Padilha da Silveira, Maria Klara Otake Hamoy, Nilton Akio Muto, Dielly Catrina Favacho Lopes, Moisés Hamoy

**Affiliations:** ^1^ Laboratory of Pharmacology and Toxicology of Natural Products, Institute of Biological Sciences Federal University of Pará Belém Pará Brazil; ^2^ Laboratory of Experimental Neuropathology, João de Barros Barreto University Hospital Federal University of Pará Belém Pará Brazil; ^3^ Center for the Valuation of Bioactive Compounds from the Amazon Federal University of Pará Belém Pará Brazil

**Keywords:** acetaminophen, brainwaves, electrocardiogram, electroencephalogram, *N*‐acetyl‐*p*‐aminophenol

## Abstract

*N*‐acetyl‐*p*‐aminophenol (APAP), or acetaminophen, is the most prescribed analgesic and antipyretic, even used in association with other drugs. Because it is easily bought in drugstores, much evidence about its toxic effects is known due to high doses or continuous use, which can lead to death in cases of intoxication. The present study aimed demonstrate the ability of APAP to change the brain and heart electrical activities, as early as significant increase in liver enzymes. Thus, a toxic dose of 1 g/kg was administered orally, and the animals were assessed every 3 h until 12 h for electroencephalography, electrocardiography and liver function data. The results showed that a toxic dose of APAP was capable of reducing brain electrical activity for the delta, theta, beta and gamma waves; however, the alpha wave activity was increased. In the heart, a reduced electrical activity was observed synchronously with the electroencephalogram. During the 12‐h follow‐up period, a significant increase in liver enzymes was observed. Taken together, we conclude that high doses of APAP can alter frequency brain oscillations and promote cardiac changes independently of liver damage in the first 12 h.

## Plain Language Summary

*N*‐acetyl‐*p*‐aminophenol (APAP), or acetaminophen or paracetamol, is the most prescribed analgesic and antipyretic in the world. However, it could cause toxic effects in high doses or with continuous use and can lead to death in cases of intoxication. This study showed that high doses of APAP (1 g/kg via oral) can alter frequency brain oscillations (brain activity) and promote cardiac changes independently of liver damage in the first 12 h in rats.

## Graphical abstract

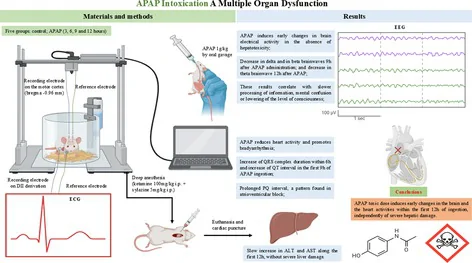

## 1 Introduction

Substance overdose and the adverse effects of this behaviour remain a daily reason for admission in intensive care units, with the potential for clinical deterioration and hemodynamic instability [1]. Somehow, *N*‐acetyl‐*p*‐aminophenol (APAP), also known as acetaminophen or paracetamol, a common analgesic and antipyretic drug easily found, presents high cases of overdose and intoxication and remains a relevant problem in the emergency department [2].

Although it is effective as an antipyretic and analgesic, its anti‐inflammatory properties are minimal, especially compared with nonsteroidal anti‐inflammatory drugs (NSAIDs). Nevertheless, APAP is more preferred to treat fever and pain than NSAIDs because its lower risk of gastrointestinal side effects (e.g., toxic ulceration and bleeding), and also, it could be better tolerated in sensitized people for allergies to NSAIDs (e.g., acetylsalicylic acid, dipyrone and ibuprofen). The APAP is usually one of the few resources for those people, and rarely, patients experience an erythematous or urticarial rash or other allergic complications [3].

APAP is an effective and safe drug at recommended doses; however, it is a dose‐dependent hepatotoxin and could cause acute liver failure (ALF) with excessive doses [4, 5]. In Western countries, the hepatotoxicity induced by high doses of APAP remains one of the main causes of ALF, as well as public health concern and ultimately for emergent liver transplantation. Annually, in the United States of America, up to 42% of hospitalizations for ALF are attributable to APAP overdose [6]. In addition, 61% of APAP‐related cases were generally related to unintentional overdose of APAP combination products prescribed by healthcare professionals [3] or the intentional overdoses used in suicide attempts [7].

Furthermore, the toxic metabolites generated during overdose produce severe organ toxicity [4, 7, 8]. Thus, ALF can produce a cerebral syndrome and hepatic encephalopathy (HE). The HE reproduces neuropsychiatric symptoms ranging from mild to severe and may lead to cerebral oedema with herniation, compression of bulbar structures and cardiorespiratory arrest [9, 10]. In addition, it can cause changes at the cellular and molecular level, such as alterations in the morphology of astrocytes and in glutamatergic pathways [9]. However, Wistar rats are more resistant to liver damage caused by APAP [11], which may be useful in demonstrating the effects of APAP on disrupting homeostasis after administration of high doses and serving as a model of APAP activity in the brain before causing severe liver damage. Therefore, the present study proposes to evaluate changes in brain oscillations caused by the acute effects of APAP on the motor cortex of Wistar rats.

## 2 Methods

### 2.1 Chemicals

The chemical substances used to conduct the present experiment were ketamine hydrochloride, obtained from the Köing Laboratory (Santana de Parnaíba, SP, Brazil); xylazine hydrochloride, obtained from the Vallée laboratory (Montes Claros, MG, Brazil); diazepam from União Química (São Paulo, SP, Brazil); lidocaine, from the Hipolabor laboratory (Sabará, MG, Brazil) for electrode implantation; and acetaminophen (drops), from the Aché Laboratório Farmacêutico SA (Guarulhos, São Paulo).

### 2.2 Animals

The research was proceeded according to the National legislation for the use and care of Animals for scientifically purpose and the Ethical Principles of the National Council for the Control of Animal Experimentation (CONCEA) with a protocol approved by the CEUA ethics committee no. 1649221220 (ID 001621) and performed according to the Animal Research: Reporting In Vivo Experiments (ARRIVE) guidelines [12]. Also, the study was conducted in accordance with the Basic & Clinical Pharmacology & Toxicology policy for experimental and clinical studies [13].

Forty‐five male Wistar rats, 12 weeks old, weighing between 200 and 230 g, from the Animal Facility of the Federal University of Pará, were housed in the Laboratory of Pharmacology and Toxicology of Natural Products, under conditions of free access to water and food ad libitum, with a room temperature of 23°C–25°C and a light–dark cycle of 12 h.

All animals in the experiments were followed up with electroencephalographic (EEG) and electrocardiographic (ECG) recordings and subsequently had a blood sample collected to assess liver function. The animals were divided into the following experimental groups (*n* = 9 animals/group): (a) control group, animals that received 0.9% saline via gavage; (b) *N*‐acetyl‐*p*‐aminophenol 3‐h group (APAP‐3h), animals that received a dose of 1 g/kg per body weight (via gavage) and were evaluated 3 h later by EEG and ECG recordings and blood collection; (c) *N*‐acetyl‐*p*‐aminophenol 6‐h group (APAP‐6h), animals that received a dose of 1 g/kg per body weight and were evaluated 6 h later by EEG and ECG recordings and blood collection; (d) *N*‐acetyl‐*p*‐aminophenol 9‐h group (APAP‐9h), animals that received a dose of 1 g/kg per body weight and were evaluated 9 h later by EEG and ECG recordings and blood collection; and (e) *N*‐acetyl‐*p*‐aminophenol group 12‐h group (APAP‐12 h), animals that received a dose of 1 g/kg per body weight and were evaluated 12 h later by EEG and ECG recordings and blood collection. All recordings lasted 3 min. After the experiment, the animals were anaesthetized with a combination of ketamine hydrochloride (80 mg/kg) and xylazine hydrochloride (10 mg/kg), and the blood was collected via cardiac puncture, followed by animal euthanasia.

### 2.3 Electroencephalography

Electrode implantation was performed as previously described in detail [14]. Briefly, for electrode implantation, the rats were anaesthetized with a combination of ketamine hydrochloride (50 mg/kg) and xylazine hydrochloride (5 mg/kg). After interruption of the corneal reflex, the animals were placed into a stereotactic frame, and the skull was exposed. Two bilateral holes were made in the skull with a dental drill. For acquisition of electroencephalographic data, stainless steel electrodes (1.0 mm diameter tip exposure) were placed on the dura mater above the frontal cortex at Bregma coordinates −0.96 mm and ±1.0 mm lateral [15]. A small screw was placed in the occipital region, and the electrodes were affixed with dental acrylic cement. On the fifth day after surgery, with the animals awake, the electrodes were connected to a data acquisition system, consisting of a high‐impedance amplifier (Grass Technologies, P511), monitored by an oscilloscope (Protek, 6510); the data were continuously digitized at a 1‐kHz rate by a computer equipped with a data acquisition board (National Instruments, Austin, TX), stored on a hard disk and processed through specialized software (LabVIEW Express). The recording electrode was located on the right side of the hemisphere, and the reference electrode on the left side. The entire experiment was carried out inside Faraday's cage.

For the analysis of electrophysiological data, the Python programming language (version 2.7) was used for offline analysis. For mathematical processing, the ‘Numpy’ and ‘Scipy’ libraries were used, and for graphing and plotting, the ‘Matplolib’ library was used. Amplitude graphs demonstrate the potential difference between the reference and recording electrodes. In the signals, 1000 samples per second were observed. Record analyses were performed up to 40 Hz, according to delta (1–4 Hz), theta (4–8 Hz), alpha (8–12 Hz), beta (12–28 Hz) and gamma (28–40 Hz).

### 2.4 Electrocardiography

The ECG activity was obtained as described by Muto et al. [16], and it was obtained from lead D‐II. To avoid excessive movement, an injection of 2.5 mg/kg (intraperitoneal) of diazepam was administered, the electrodes were made in a non‐conjugated way, the reference electrode was placed under the right axilla (0.5 cm) and the recording electrode was fixed in the tenth intercostal space (3.5 cm), below the left axilla, obeying the register vector. Each record lasted 5 min, and the analysed data were heart rate (beats per minute), R‐R interval (ms), P‐Q interval (ms), QRS duration (ms) and QT interval (ms).

### 2.5 Liver Function

After the ECG recordings, a sample of blood was collected through cardiac puncture for analysis of liver function. The blood was stored in a flask containing EDTA for plasma removal, followed by analysis by the Labtest Kit performed by fabricant instructions (Lagoa Santa, MG, Brazil) for alanine aminotransferase (ALT or GPT [glutamate pyruvate transaminase]) and aspartate aminotransferase (AST or GOT [glutamic oxaloacetic transaminase]) using spectrophotometry calibrated in absorbance of 340 nm.

### 2.6 Statistical Analysis

All data were acquired and evaluated through a double‐blind study. The normality of the data variances was verified using the Kolmogorov–Smirnov test. Data distributed normally and with homogeneous variances were analysed by a one‐way ANOVA followed by Tukey's test for multiple comparisons. Otherwise, data that did not pass normality were analysed using a Kruskal–Wallis's test, followed by Dunn's test for multiple comparisons. All data are presented as the mean and standard deviation (SD), and *F* or *H* and *p*‐values are included when pertinent. A *p* < 0.05 significance level was considered for all analyses. The data were analysed using GraphPad Prism, version 9 (GraphPad Software Inc., San Diego, CA, USA).

## 3 Results

### 3.1 APAP Alters the Electrical Pattern of the Brain

In the EEG tracing of the control group, a characteristic amplitude lower than 0.05 mV and the frequencies observed in the spectrogram presented higher energy intensity below 10 Hz (Figure 1A). For 3 and 6 h after administration of APAP, the EEG patterns were similar to the control (Figure 1B–C). Although 9 and 12 h after administration of APAP, the EEG tracing was lower than the control at frequencies of 28–40 Hz (gamma), some irregularities in the tracing with amplitude variations were also observed (Figure 1D–E).

**FIGURE 1:**
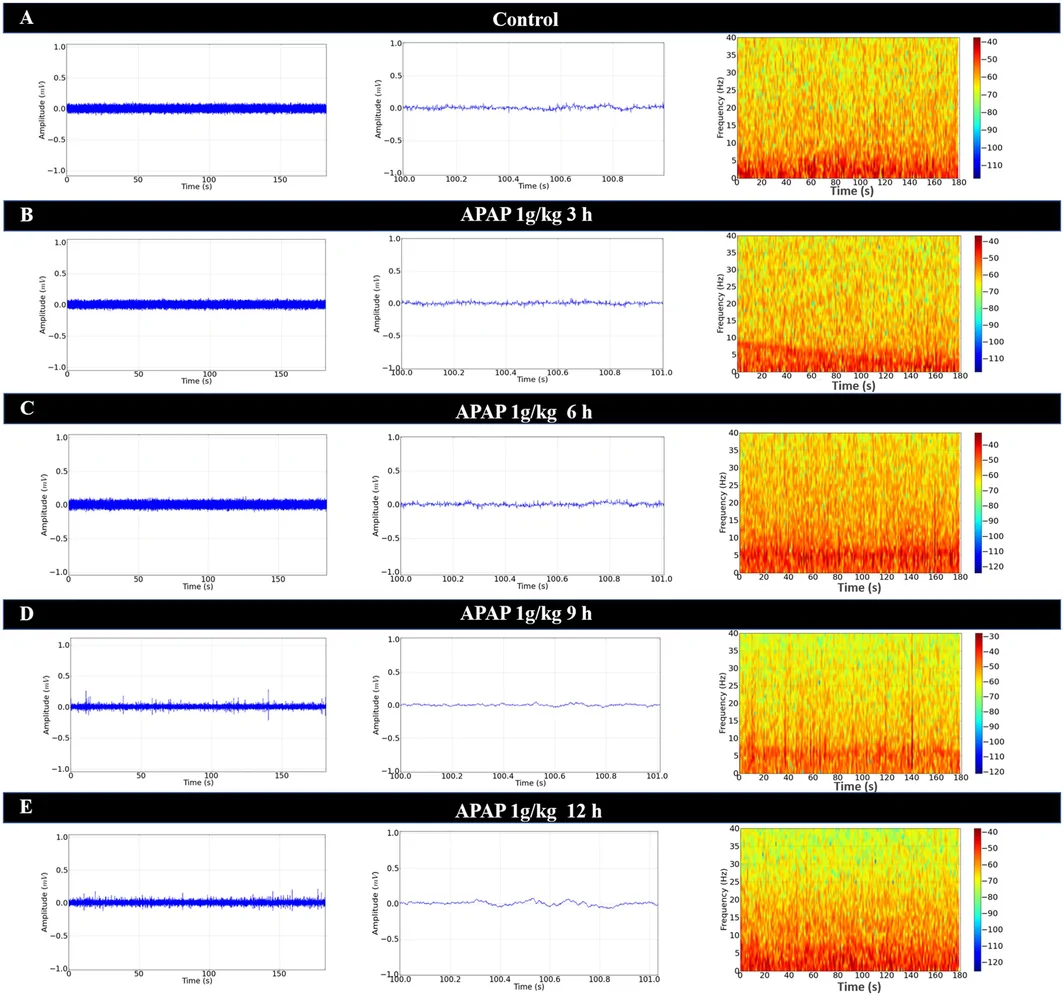
Electroencephalographic recordings in the control group and animals treated with APAP. Three minutes of EEG trace (left); representative fragment of 1 s from EEG tracing (centre); and spectrogram of the frequencies (right). (A) Control group. (B) APAP group 3 h. (C) APAP group 6 h. (D) APAP group 9 h. (E) APAP group 12 h. APAP: *N*‐acetyl‐*p*‐aminophenol.

In the total power analysis (*F*_(4,40)_ = 22.81, *p* < 0.0001), the distribution of the spectral power revealed lower amplitude for times 9 h (0.088 ± 0.012 mV^2^/Hz × 10^−3^) and 12 h (0.065 ± 0.019 mV^2^/Hz × 10^−3^) after APAP administration in comparison with control (0.142 ± 0.012 mV^2^/Hz × 10^−3^), 3 h (0.132 ± 0.023 mV^2^/Hz × 10^−3^) and 6 h (0.117 ± 0.003 mV^2^/Hz × 10^−3^; *p* < 0.05 for all comparisons; Figure 2A).

**FIGURE 2:**
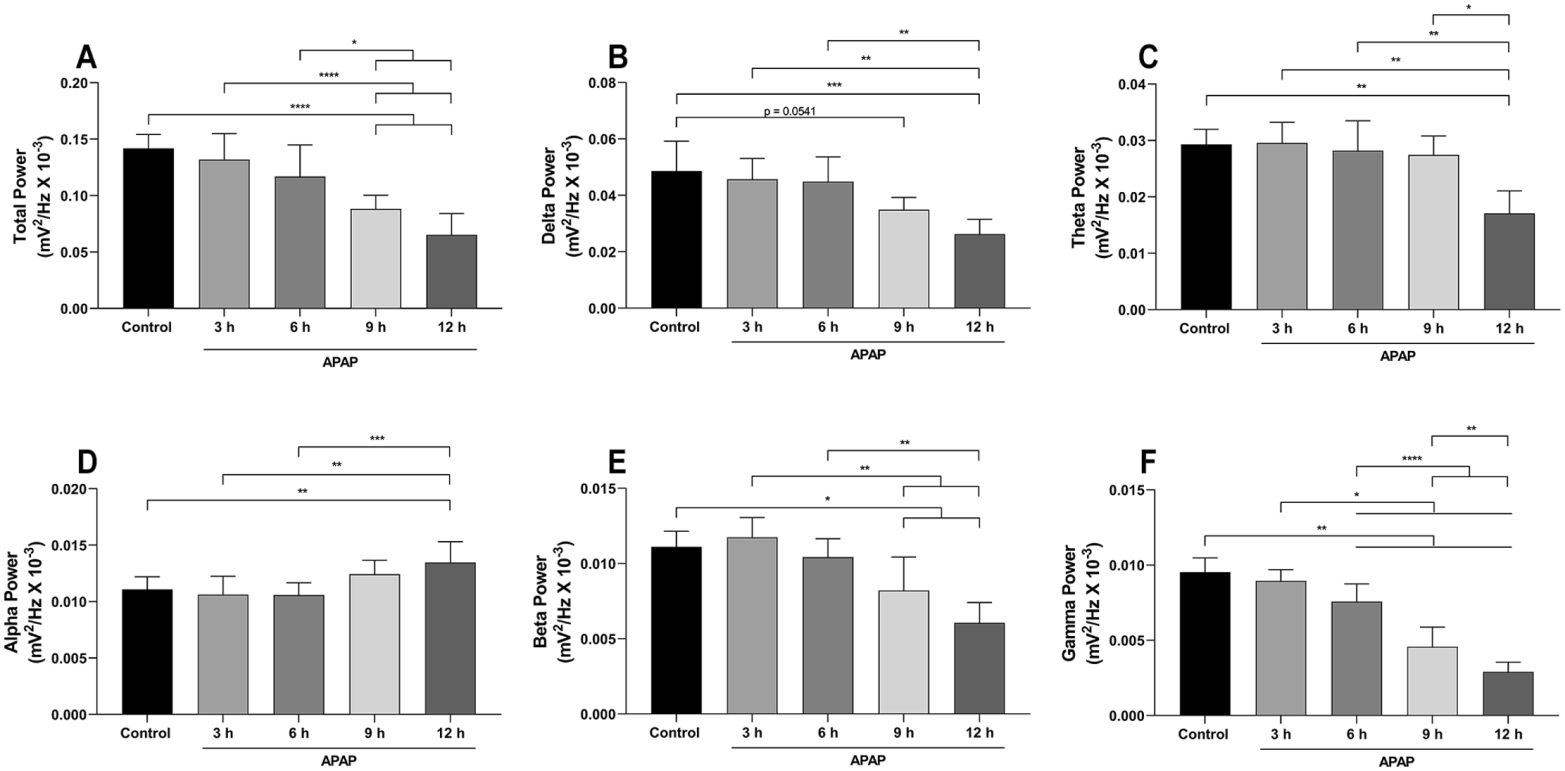
Decomposition of the brainwaves in the control animals and APAP‐treated. (A) Linear frequency distribution of the total power. (B) Linear frequency distribution of the delta waves. (C) Linear frequency distribution of the theta waves. (D) Linear frequency distribution of the alpha waves. (E) Linear frequency distribution of the beta waves. (F) Linear frequency distribution of the gamma waves. The data are expressed as the mean ± SD (*n* = 9 animals per group; *p < 0.05, **p < 0.01, ***p < 0.001 and ****p < 0.0001). APAP: *N*‐acetyl‐*p*‐aminophenol.

For the delta wave (1–4 Hz; H(4) = 26.05, *p* < 0.0001), animals assessed 12 h after APAP administration (0.026 ± 0.005 mV^2^/Hz × 10^−3^) presented lower delta power in comparison with control (0.047 ± 0.011 mV^2^/Hz × 10^−3^), APAP 3 h (0.046 ± 0.007 mV^2^/Hz × 10^−3^) and APAP 6 h (0.045 ± 0.009 mV^2^/Hz × 10^−3^) groups (*p* < 0.01 for all comparisons; Figure 2B). Similarly, the analysis for the theta wave (4–8 Hz; H(4) = 21.03, *p* = 0.0003) showed power brainwave reduction between APAP 12 h (0.017 ± 0.004 mV^2^/Hz × 10^−3^) and all other groups (control: 0.029 ± 0.003 mV^2^/Hz × 10^−3^; APAP 3 h: 0.030 ± 0.004 mV^2^/Hz × 10^−3^; APAP 6 h: 0.028 ± 0.005 mV^2^/Hz × 10^−3^; and APAP 9 h: 0.027 ± 0.003 mV^2^/Hz × 10^−3^; *p* < 0.05 for all comparisons; Figure 2C).

Differently than was shown to delta and theta brainwaves, a significant increase of amplitude was found in the distribution of alpha waves (*F*_(4,40)_ = 7.159, *p* = 0.0002) at 12 h (0.013 ± 0.002 mV^2^/Hz × 10^−3^) after APAP administration in comparison with control (0.011 ± 0.001 mV^2^/Hz × 10^−3^), APAP 3 h (0.010 ± 0.002 mV^2^/Hz × 10^−3^) and APAP 6 h (0.011 ± 0.001 mV^2^/Hz × 10^−3^) groups (*p* < 0.01 for all comparisons; Figure 2D).

In the case of the beta wave frequency (12–28 Hz; H(4) = 31.06, *p* < 0.0001), there was a significant reduction of beta power over time treatment, with the lowest response being recorded in the APAP 9 h (0.008 ± 0.002 mV^2^/Hz × 10^−3^) and APAP 12 h (0.006 ± 0.001 mV^2^/Hz × 10^−3^), and both groups were significantly lower than the control (0.011 ± 0.001 mV^2^/Hz × 10^−3^; *p* < 0.05 for all comparisons) and APAP 3 h (0.012 ± 0.001 mV^2^/Hz × 10^−3^; *p* < 0.01 for all comparisons) groups. In addition, a difference was observed between APAP 6 h (0.010 ± 0.001 mV^2^/Hz × 10^−3^) and APAP 12 h (*p* < 0.01; Figure 2E).

For the gamma waves (*F*_(4,40)_ = 73.22, *p* < 0.0001), a significant variation was observed among groups above 6 h after APAP administration. Here, the lower data were recorded in the APAP 6 h (0.0075 ± 0.0011 mV^2^/Hz × 10^−3^), APAP 9 h (0.0046 ± 0.0013 mV^2^/Hz × 10^−3^) and APAP 12 h (0.0029 ± 0.0006 mV^2^/Hz × 10^−3^), and all groups were significantly lower than the control (0.0095 ± 0.0009 mV^2^/Hz × 10^−3^; *p* < 0.05 for all comparisons) and APAP 3 h (0.0089 ± 0.0007 mV^2^/Hz × 10^−3^; *p* < 0.01 for all comparisons). Also, a difference was observed between APAP 6 h vs. APAP 9 h and vs. APAP 12 h (*p* < 0.0001); and APAP 9 h vs. APAP 12 h (*p* < 0.01; Figure 2F).

### 3.2 APAP Alters the Electrical Pattern of the Heart

The cardiac activity of the control group was recorded lasting 10 s (Figure 3A) and after amplification in 1 s, it can be observed the sinus rhythm, showing all cardiac triggers, with activity represented by the P wave, ventricular activity represented by the QRS complex and ventricular repolarization shown by T wave (Figure 3B).

**FIGURE 3:**
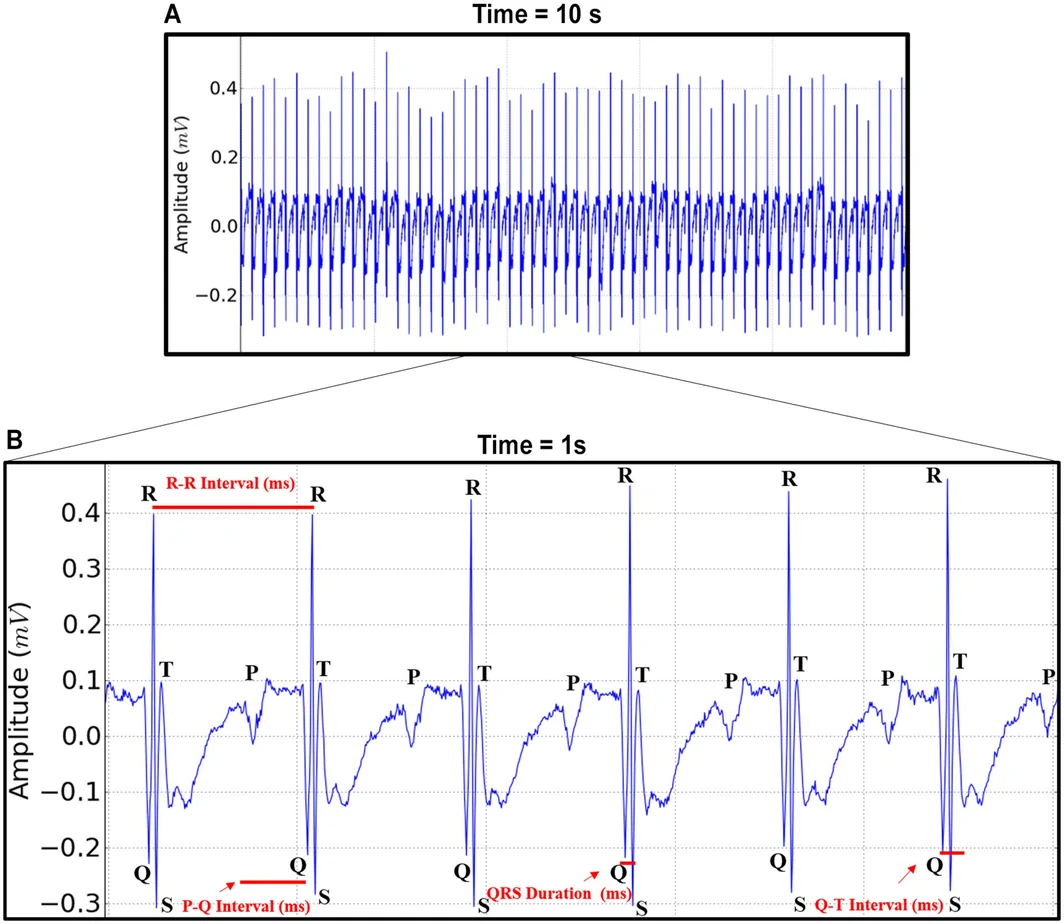
Control electrocardiogram in lead D‐II in the rat. (A) Recording in 10 s of the ECG. (B) Enlargement of the record in 1 s and in the red lines represents the intervals to be analysed: RR interval (ms), PQ interval (ms), QT interval (ms), QRS complex duration (ms).

In the groups that received APAP and were evaluated 3 and 6 h after gavage, it can be observed that sinus rhythm and all cardiac outbreaks were similar to the control group (APAP 3H, Figure 4A–B; APAP 6H, Figure 4C–D). However, 9 h after APAP administration, although the sinus rhythm was similar to the control group, a decrease in cardiac activity was observed (APAP 9H, Figure 4E–F); whereas the APAP 12 h group showed decreased cardiac activity, with sinus rhythm (APAP 12H, Figure 4G–H). One of the changes in the recordings was the shape of the T wave, which showed greater opening and irregularities.

**FIGURE 4:**
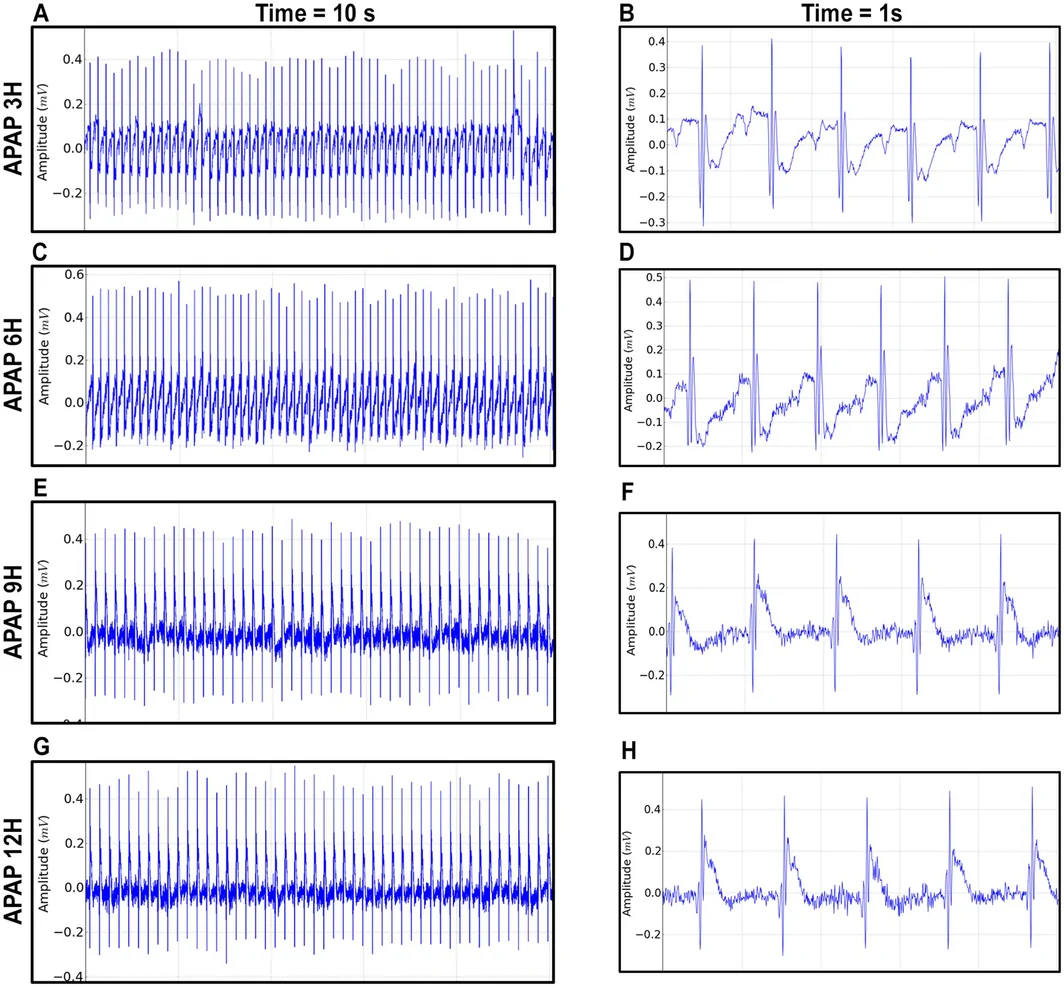
Electrocardiogram in lead D‐II in the rat with 10 s of ECG trace (left) and representative fragment of 1 s from ECG tracing (right). (A–B) APAP 3 h group. (C–D) APAP 6 h group. (E–F) APAP 9 h group. (G–H) APAP 12 h group.

The heart rate (*F*_(4,40)_ = 48.92, *p* < 0.0001) was reduced from 9 h after APAP administration. Thus, APAP 9 h and 12 h presented averages of 307.8 ± 18.56 and 298.7 ± 14.45 bpm, respectively, and both groups were significantly lower than control (358.3 ± 10.31 bpm), APAP 3 h (363.1 ± 98.54) and APAP 6 h (351.1 ± 10.54 bpm) (*p* < 0.0001 for all comparisons; Figure 5A).

**FIGURE 5:**
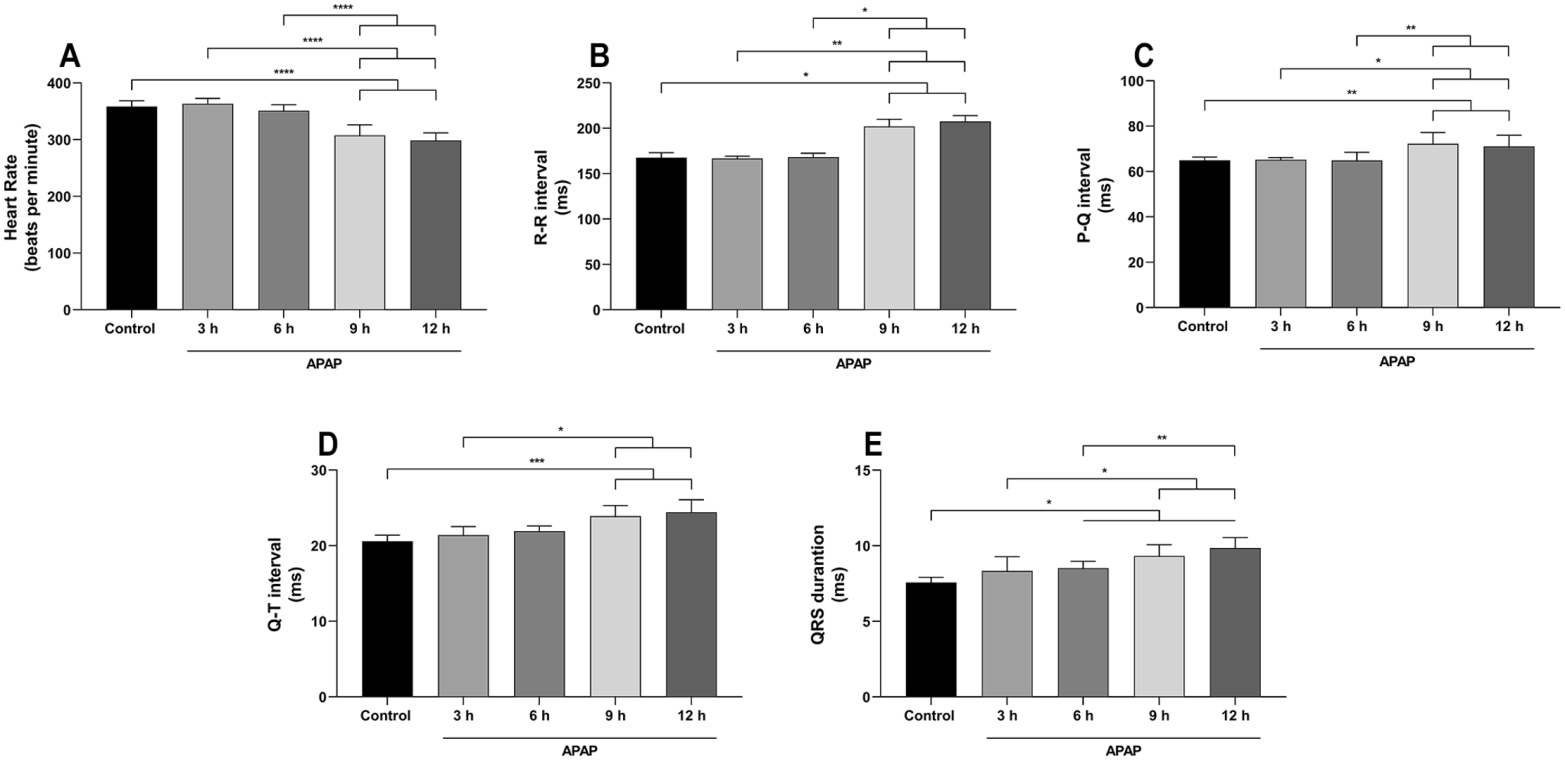
Quantitative representation of the ECG. (A) Heart rate means (bpm) recorded in the control groups, 3 h, 6 h, 9 h and 12 h after administration of APAP. (B) Evaluation of the mean RR intervals (ms). (C) Evaluation of mean PQ intervals. Assessment of mean Q‐T intervals (ms). (E) Assessment of mean QRS complex duration (ms). The data are expressed as the mean ± SD (*n* = 9 animals per group; *p < 0.05, **p < 0.01, ***p < 0.001 and ****p < 0.0001).

The R‐R interval [H(4) = 33.76, *p* < 0.0001] was increased for 9 (202.2 ± 7.76 ms) and 12 h (207.6 ± 6.64 ms) after APAP administration in comparison with control (167.8 ± 5.33 ms; *p* < 0.05 for all comparisons), APAP 3 h (166.7 ± 2.78 ms; *p* < 0.01 for all comparisons) and APAP 6 h (168.3 ± 4.21 ms; *p* < 0.05 for all comparisons; Figure 5B). Similarly, P‐Q interval (*F*_(4,40)_ = 9.134, *p* < 0.0001) was also increased after 9 h, showing similar statistical differences to P‐Q interval (control: 64.94 ± 1.44 ms; APAP 3 h: 65.19 ± 0.96 ms; APAP 6 h: 64.89 ± 3.62 ms; APAP 9 h: 72.21 ± 5.03 ms; APAP 12 h: 71.08 ± 4.96 ms; Figure 5C).

For the Q‐T interval [H(4) = 27.73, *p* < 0.0001], the control (20.59 ± 0.80 ms), APAP 3 h (21.40 ± 1.15 ms) and APAP 6 h (21.91 ± 0.70 ms) groups did not show statistical differences among each other. On the other hand, APAP 9 h (23.89 ± 1.41 ms) and APAP 12 h (24.41 ± 1.68 ms) groups were different from control (*p* < 0.001, for all comparisons) and APAP 3 h (*p* < 0.05, for all comparisons) groups (Figure 5D).

In the analysis of QRS duration (*F*_(4,40)_ = 15.87, *p* < 0.0001), a significant variation was observed among groups above 6 h after APAP administration. Thus, the highest data were observed from APAP 6 h (8.52 ± 0.45 ms), APAP 9 h (9.33 ± 0.75 ms) and APAP 12 h (9.86 ± 0.69 ms), and all groups were significantly lower than the control (7.57 ± 0.34 ms; *p* < 0.05 for all comparisons). Also, there was a difference between APAP 3 h (8.33 ± 0.95 ms) and APAP 9 h (*p* < 0.05) and APAP 12 h (*p* < 0.001); and APAP 6 h in comparison with APAP 12 h (*p* < 0.01; Figure 5E).

### 3.3 APAP Induces Alterations in Liver Enzymes Early

The biochemical analyses showed that ALT activity (*F*_(4,40)_ = 8.461, *p* < 0.0001) for the control (36.47 ± 7.88 U/L), APAP 3 h (39.80 ± 12.67 U/L) and APAP 6 h (47.29 ± 5.30 U/L) groups did not reveal significant differences between each other. On the other hand, both APAP 9 h (54.62 ± 6.60 U/L) and APAP 12 h (52.67 ± 6.14 U/L) groups showed higher ALT levels in comparison with control (*p* < 0.01, for both comparisons) and APAP 3 h (*p* < 0.05, for both comparisons; Figure 6A).

**FIGURE 6:**
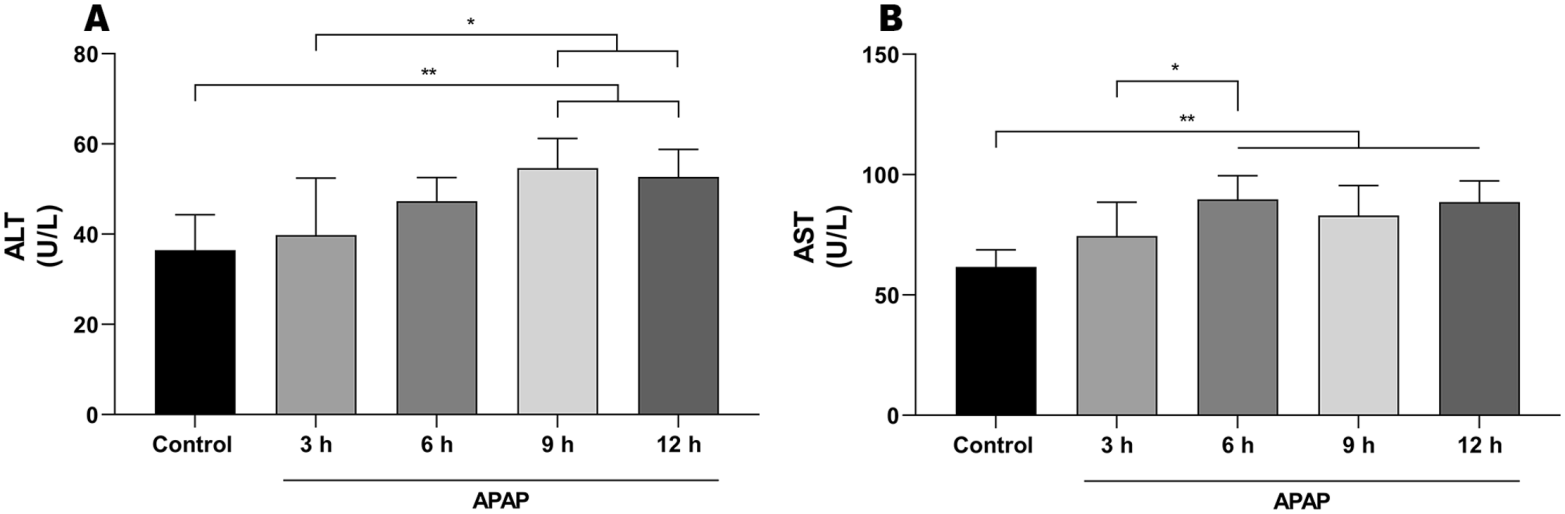
The mean ALT (A) and AST (B) activities (U/L) in the control and APAP groups. The data are expressed as the mean ± SD (*n* = 9 animals per group; *p < 0.05, **p < 0.01). APAP: *N*‐acetyl‐*p*‐aminophenol.

For the AST (*F*_(4,40)_ = 10.48, *p* < 0.0001), a significant difference was observed among groups above 6 h after APAP administration (APAP 6 h: 89.69 ± 9.90 U/L; APAP 9 h: 83.05 ± 12.51 U/L; and APAP 12 h: 88.57 ± 8.97 U/L), and all groups were significantly higher than the control (61.60 ± 7.16 U/L; *p* < 0.01 for all comparisons). Also, it was observed between APAP 3 h (74.48 ± 14.13 U/L) and APAP 6 h (*p* < 0.05; Figure 6B).

## 4 Discussion

Brain alterations caused by APAP intoxication may have a primary and secondary or multifactorial cause. It is believed that ammonia accumulation may be one of the main factors responsible for the development of HE. This occurs because there is an arrest in the urea cycle in the liver due to acute liver necrosis, which causes high levels of NH_4_^+^ to accumulate in the plasma [17]. The NH_4_^+^ inside astrocytes is transformed into glutamate by conjugation with alpha‐ketoglutarate. This leads to the formation of osmotic stress, which is harmful to these cells, and also the release of high levels of glutamate, leading to glutamatergic toxicity, oxidative stress and activation of pro‐inflammatory mediators [17, 18].

In addition, changes in brain dynamics can also be caused by the action of acetaminophen directly on the brain, when it crosses the blood–brain barrier and is uniformly distributed [19, 20]. The majority of APAP hepatic metabolism (90%) is delivered by UDP‐glucuronosyltransferases (UGT) and sulfotransferases (SULT), which results in drug conversion to glucuronidated and sulphated non‐toxic metabolites excreted in urine [21]. However, 10% of APAP is eliminated via cytochrome P450 (CYP) isoforms involved in hepatic metabolism, with emphasis on CYP2E1, which is responsible for the formation of the highly reactive metabolite NAPQI (*N*‐acetyl‐*p*‐benzoquinone imine). Subsequently, in physiological conditions, NAPQI is conjugated by hepatic glutathione (GSH) to form non‐toxic mercaptate and cysteine compounds that undergo urinary excretion [22]. With APAP overdose, the glucuronidation and sulfonation pathways become saturated, which deviates the drug metabolism towards CYP2E1 metabolism, resulting in GSH depletion and accumulation of toxic NAPQI [7, 23].

CYP2E1 is also expressed in the brain, which may explain the electrical changes in the brain without the presence of acute hepatic necrosis. This isoform is present in important regions, such as the cortex, hippocampus, cerebellum and brainstem, which suggests that acetaminophen can be metabolized by brain cells and produce NAPQI in situ [24, 25, 26]. Studies have reported a decrease in GSH content in the rat brain of animals treated with a toxic dose of APAP (3 g/kg), with the most affected regions corresponding to the hypothalamus, raquideal bulb and cerebral cortex. These studies also demonstrated that APAP overdose induced oxidative stress in the rat brain, as shown by the increase of lipid peroxidation (expressed as malondialdehyde accumulation) and the reduction of antioxidant molecules (GSH, ascorbic acid and superoxide dismutase) [27, 28]. Additionally, APAP intoxication leads to an elevation of neuronal CYP2E1 enzymatic activity and protein levels, resulting in neuronal cell death through mitochondrial‐mediated mechanisms [29]. Thereby, there is supporting evidence indicating that brain alterations in APAP‐induced hepatotoxicity involve CYP2E1 and NAPQI accumulation to the detriment of glucuronidation and sulfonation pathways. However, few studies have concentrated on investigating the effects on the electrical dynamics of the brain in high doses of acetaminophen.

In our study, we decided to evaluate EEG and ECG changes in the first 12 h after APAP acute overdose, considering that the time to initiate therapy with the antidote N‐acetylcysteine is a determinant factor in presuming drug hepatotoxicity development and severity [22]. Symptoms of acute intoxication include nausea, vomiting, malaise, lethargy and diaphoresis and appear within 24 h, while AST and ALT levels start to increase in 8–12 h. Within 72–96 h after APAP overdose, AST and ALT significantly increase (> 3000 IU/L) in conjunction with jaundice, encephalopathy, coagulopathy, lactic acidosis and renal failure [22]. This stage is marked by maximal liver injury, which implies poor response to treatment, guarded prognosis and high risk of mortality. Thus, we decided to monitor brain and heart function during the acute phase, considering the critical time for therapeutic intervention before the development of liver failure, preventing multi‐organ failure and other complications.

It has been well known that ALF can increase toxic metabolites and induce behavioural disorders. Isobe‐Harima et al. [30] described a significant increase in blood ammonia, glucose levels and prothrombin time within the first 3 h of 1‐g/kg APAP intoxication in Wistar rats, as well as a boost in glutamate and aspartate neural amino acids in the hippocampus from 4 h [30]. Therefore, possibly there are early changes in electrical brain activity not very well explored by the current literature and that can be easily assessed by a non‐invasive tool as EEG.

In this work, we are one of the few groups to demonstrate the electroencephalographic effects of acute acetaminophen overdose. The classic EEG finding in patients with severe liver damage is triphasic waves, although these are no longer considered specific for liver failure and may result from a diverse group of metabolic disorders, and also, electrocerebral silence has been shown previously [31, 32]. We observed in our data that there are changes in the elements of the graph in the EEG, with a more intense onset 9 h after treatment. There was a decrease in the power of brain oscillations in delta and beta, which may result in slower processing of information, mental confusion or lowering of the level of consciousness, although we cannot presume that these findings correspond to HE, given the absence of a severe increase in aminotransferases [18, 33].

EEG changes in early HE usually include a slowing of the posterior dominant rhythm, followed by a gradual slowing of the background with the appearance of theta and delta activity [34]. Frontal intermittent rhythmic delta activity can appear even in the presence of the posterior dominant rhythm [33]. The results show that 9 h after treatment there was a decrease in power in delta brain oscillations and 12 h after treatment there was a decrease in power in theta brain oscillations. However, there was no severe liver injury that would indicate HE.

Patients intoxicated with acetaminophen have higher serum levels of alanine aminotransferase (ALT), aspartate aminotransferase (AST) and serum creatinine than those with ALF from other causes [3]. Of those who met the criteria, 77% had detectable acetaminophen levels in their serum, and 91% had ALT levels higher than 1.000 IU/L [3]. Our data show that elevations in liver enzymes occur gradually after contact with an acute toxic dose; however, brain and heart activities are already altered, although the gradual and time‐dependent increase observed in our study for ALT and AST is within normal parameters for Wistar rats [35].

Using a mouse model is more clinically convenient than a rat one to study hepatotoxicity, since there are important differences between these species concerning APAP metabolism. First, McGill et al. [21] described a delay in GSH depletion and APAP‐protein adduct formation in rats compared to mice treated with toxic doses of APAP (1 g/kg for rats and 300 mg/kg for mice). The same work also reported oxidative stress (increase in glutathione disulfide levels, peroxynitrite formation and JNK phosphorylation in liver tissue) in mice treated with APAP, but not in rats, so this evidence suggests rats are resistant to severe liver injury [21]. In addition, APAP doses that far exceed the lethal doses (LD_50_) for mice cause only minimal necrosis in rat liver [36], which may limit the mouse model to approach the brain changes directly induced by APAP intoxication, given that these alterations could be easily confused with HE.

Sustained consumption of acetaminophen at therapeutic doses (4 g per day) can lead to asymptomatic increases in blood concentrations of hepatic aminotransferases after more than 4 days of consumption [37]. These data corroborate our results, since the elevations of liver enzymes change slowly in the case of ingestion of high doses.

Electrolyte disturbance may occur in APAP poisoning secondary to gastrointestinal loss due to recurrent vomiting episodes and results in hypovolemia, hyponatremia and hypokalaemia. Tubular affection in acute kidney injury induced by APAP is another cause of sodium and potassium loss [38], which can promote fatal arrhythmias. For this reason, heart electrical activity monitoring is crucial.

Our data demonstrated that acetaminophen at a toxic dose caused ECG changes 6 h after contact. In our study, APAP provoked bradycardia and prolongation of atrioventricular (AV) conduction (expressed as P‐Q interval rise) 9 h after drug administration, which correlates with AV block, also found in other substances intoxication, such as beta‐adrenergic antagonists and calcium channel blockers [39]. Moreover, acetaminophen promoted a Q‐T interval increase in the first 9 h and a QRS duration widening within 6 h, findings that correspond to sodium channel blockade and potassium channel blockade, respectively, which lead to ventricular depolarization delay and myocardial slowed repolarization [39]. Acetaminophen has direct toxic effects on the myocardium, and cardiotoxicity can be immediate or delayed. According to a study by Jacob et al. [40], acetaminophen can deplete sulfhydryl groups, interfering with nitric oxide production and thus leading to coronary ischemia [40]. In addition, sulfhydryl deficit may interfere with endothelium‐derived vascular relaxing factor, causing functional coronary ischemia. However, there was an evolution of the clinical condition in synchrony with the electroencephalographic and ECG effects.

Our results demonstrated alterations in the power of brain oscillations prior to the biochemical elevation of ALT and AST, suggesting that the central nervous system is highly sensitive to initial metabolic insults, even before the consolidation of severe liver damage. The reduction in the power of beta and gamma bands, accompanied by an increase in alpha power, indicates a disorganization of synchronous cortical activity. In the behavioural assessment, the decrease in beta and gamma power is associated with lethargic states, disorganization of the sleep–wake cycle with a prevalence of drowsiness [41, 42]. No behavioural alterations such as incoordination, sleep–wake cycle, exploratory and motor alterations were observed, and cognitive assessments were not evaluated, which is a limitation of our work.

Based on the data presented, we demonstrated that acetaminophen in toxic doses alters the components of electroencephalographic and ECG recordings in rats; these alterations evolve rapidly and independently of liver injury. Thereby, these results are important and have prognostic value for demonstrating the early alterations in brain and heart electrical activity promoted directly by APAP out of the context of HE, since this syndrome is complex and involves multiple factors, including hyperammonaemia and systemic inflammation, which contribute to cerebral dysfunction and could be confused with APAP intoxication effects in these organs.

## Author Contributions

A.L.M.S., A.O.H., C.A.P. and M.K.O.H.: acquisition and investigation. L.G.R.F., L.O.F., E.P.S. and N.A.M.: formal analysis and interpretation of data for the work. L.O.F., D.C.F.L. and M.H.: produced the figures and tables. D.C.F.L. and M.H.: conceptualization, design, writing the paper and revising the manuscript critically. All the authors have read and approved the final version of the manuscript.

## Funding

This work was supported by Pró‐Reitoria de Pesquisa e Pós‐Graduação, Universidade Federal do Pará.

## Conflicts of Interest

The authors declare no conflicts of interest.

## Data Availability

Data will be made available on request.
